# Guided Eruption of Bilateral Palatally Impacted Maxillary Canines Using a Ballista Spring: A Case Report

**DOI:** 10.7759/cureus.115563

**Published:** 2026-08-31

**Authors:** Pratibha Saini, Virendra Vadher, Gangesh Bahadur Singh, Chhaya Barapatre, Neha Sahu

**Affiliations:** 1 Department of Orthodontics and Dentofacial Orthopaedics, Government Dental College and Hospital, Raipur, IND; 2 Department of Periodontology and Implantology, Government Dental College and Hospital, Raipur, IND

**Keywords:** ballista spring, bilateral impacted canines, fixed orthodontic appliance, guided eruption, impacted tooth, maxillary canine, orthodontic management, orthodontic traction, palatal impaction, surgical exposure

## Abstract

Palatally impacted maxillary canines are a relatively frequent developmental defect that can cause functional, aesthetic, and periodontal issues in cases of inaction. To reposition the impacted teeth into the dental arch and avoid complications related to them, early detection and proper orthodontic treatment are necessary. This case report presents the orthodontic management of a patient with bilateral palatally impacted maxillary canines. After detailed clinical and radiographic assessment, it was decided to apply the treatment plan that included surgical exposure of the impacted canines and orthodontic traction with the help of a ballista spring using a fixed orthodontic appliance. The ballista springs available offer directional and controlled forces of eruption, which allow gradual movement of the affected canines to their respective locations within the dental arch. Periodontal health was preserved through regular activation and monitoring, which enabled gradual alignment and integration of the canines into the occlusion. The effective result proves that the ballista spring is a useful and flexible aid to orthodontic treatment of palatally impacted maxillary canines, which allows control of the application of forces and promotes eruption. Proper surgical exposure, careful treatment planning, and controlled orthodontic forces coupled with regular follow-ups are critical in ensuring positive functional and aesthetic results.

## Introduction

Impaction of maxillary canines is a relatively common developmental anomaly and poses a significant challenge in orthodontic care. The maxillary canine plays a crucial role in smile aesthetics, arch integrity, and functional occlusion; therefore, preservation and orthodontic alignment of an impacted canine are desirable whenever feasible [1,2]. Impacted maxillary canines may lead to several complications, including delayed eruption, displacement or resorption of adjacent teeth, cystic changes, arch discrepancies, and aesthetic as well as functional concerns, further emphasizing the importance of timely diagnosis and appropriate management [1,3]. The etiology of canine impaction is multifactorial and involves arch-length deficiency, prolonged retention of the deciduous canine, abnormal tooth-bud position, ankylosis, cystic lesions, and other local factors [1,3]. Among maxillary canine impactions, palatal impaction constitutes a substantial proportion, and bilateral involvement further increases the complexity of treatment [2,3].

Accurate localization of the affected tooth is essential for appropriate treatment planning. Conventional radiographs provide information regarding the position and angulation of the teeth, whereas cone-beam computed tomography (CBCT) enables 3D localization and assessment of the relationship between the impacted canine and surrounding structures [4,5]. Treatment usually entails surgical exposure followed by spontaneous eruption or orthodontic traction, depending on the location and eruption potential of the tooth [6]. For palatally impacted canines, minimally invasive exposure techniques have been described to provide adequate access for orthodontic attachment while minimizing unnecessary surgical trauma. Cheng and Chang described a simplified open-window technique using electrocautery, which provides localized soft-tissue removal, hemostasis, and improved surgical visibility [7].

Following surgical exposure, successful orthodontic traction requires controlled force application, appropriate force direction, and adequate anchorage. Various traction methods have been used for impacted canines; however, the choice of auxiliary depends on the position and orientation of the impacted tooth and the desired direction of movement. The ballista spring, introduced by Jacoby, is a useful auxiliary for applying controlled eruptive forces to impacted teeth [8]. Its design allows modification of the direction of traction according to the position of the impacted canine, making it particularly suitable when controlled and directed movement is required. Previous reports have demonstrated the successful use of ballista springs for the management of palatally impacted maxillary canines, including bilateral impactions [9].

The present case highlights a combined and minimally invasive treatment approach involving CBCT-guided localization, bone sounding for precise surgical planning, electrosurgical exposure, and controlled orthodontic traction using ballista springs. By integrating accurate 3D localization with a conservative surgical technique and a biomechanically controlled method of traction, this approach aims to facilitate effective management of bilateral palatally impacted maxillary canines while minimizing surgical trauma. The present case describes the management of bilateral palatally impacted maxillary canines using a CBCT- and bone-sounding-guided minimally invasive electrosurgical exposure technique followed by orthodontic relocation using ballista springs.

## Case presentation

A 21-year-old female patient reported to the Department of Orthodontics, Government Dental College and Hospital, Raipur, in September 2024 with a chief complaint of retained upper milk teeth in the anterior region and an unaesthetic smile. The patient expressed dissatisfaction with her smile and reported feeling embarrassed while smiling because of the appearance of her anterior teeth. Intraoral examination revealed retained deciduous maxillary canines with clinically unerupted permanent maxillary canines (Figure 1). The patient presented with Angle’s Class I malocclusion on a Class I maxillomandibular skeletal base, deep bite with bilateral palatally impacted maxillary canines. Palatal prominences were not evident in the canine regions. Mild mandibular anterior crowding was also noted. Model analysis revealed a reduced arch perimeter in the maxillary arch. Ashley Howe’s index indicated a non-extraction treatment approach. Radiographic examination using orthopantomogram (OPG) and CBCT confirmed the presence of bilateral palatally impacted maxillary canines associated with over-retained deciduous maxillary canines. The alpha angle of the impacted maxillary canine was 29° on the right side and 40° on the left side (Figures 2-4), indicating the differing angulations of the impacted canines and providing an objective assessment of their position prior to surgical exposure and orthodontic traction. Based on the clinical, model, and radiographic findings, a non-extraction fixed orthodontic treatment was planned using a 0.022-inch McLaughlin, Bennett, and Trevisi (MBT) appliance. The treatment objectives were to create and maintain adequate space for the impacted canines, facilitate their surgical exposure and subsequent orthodontic relocation into the dental arch, correct mandibular anterior crowding, achieve ideal overjet and overbite, and obtain a well-balanced smile and harmonious soft-tissue profile. 

**Figure 1:**
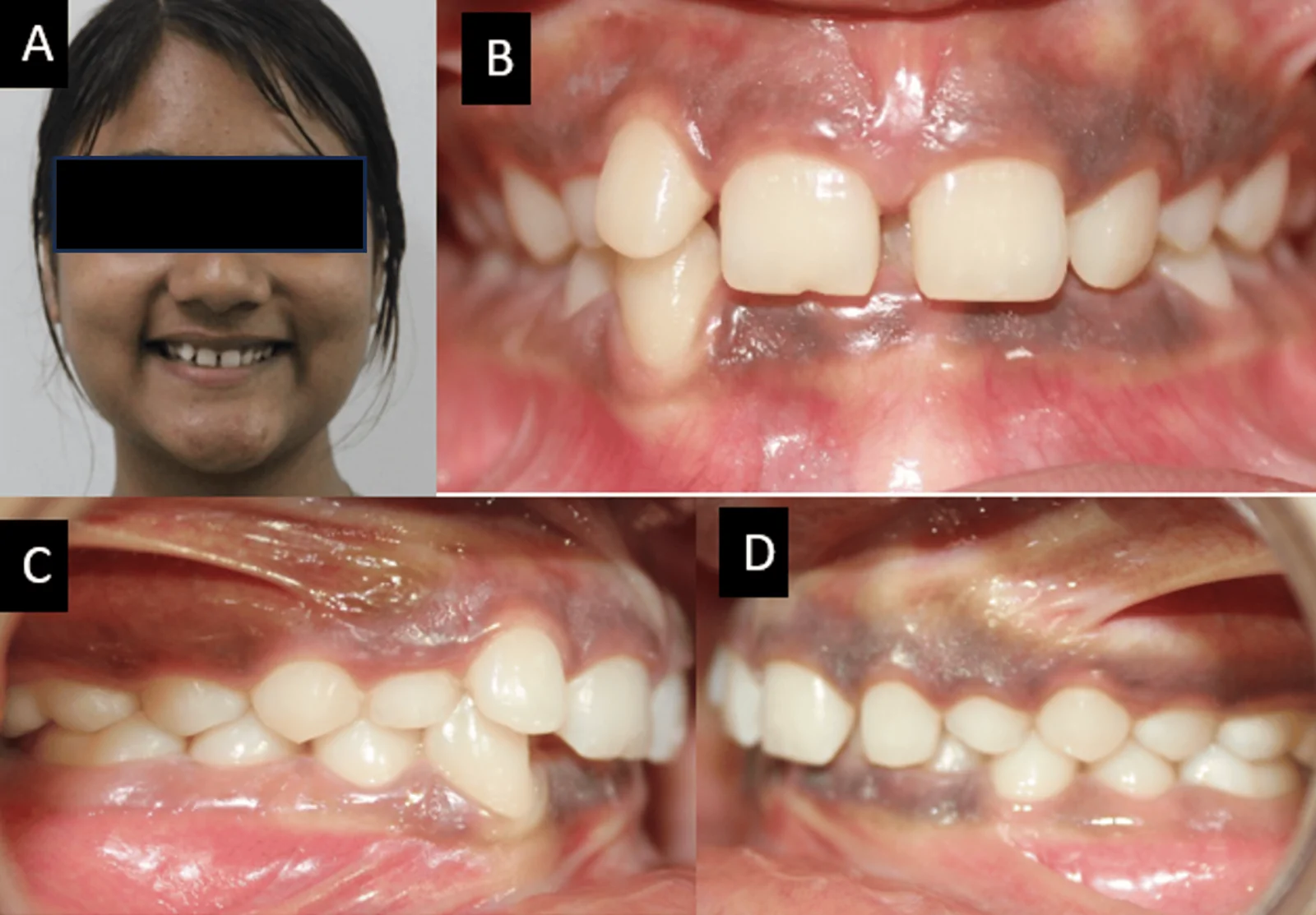
(A,B) Pre-treatment smile of patient. (C,D) Pre-treatment right and left lateral views.

**Figure 2:**
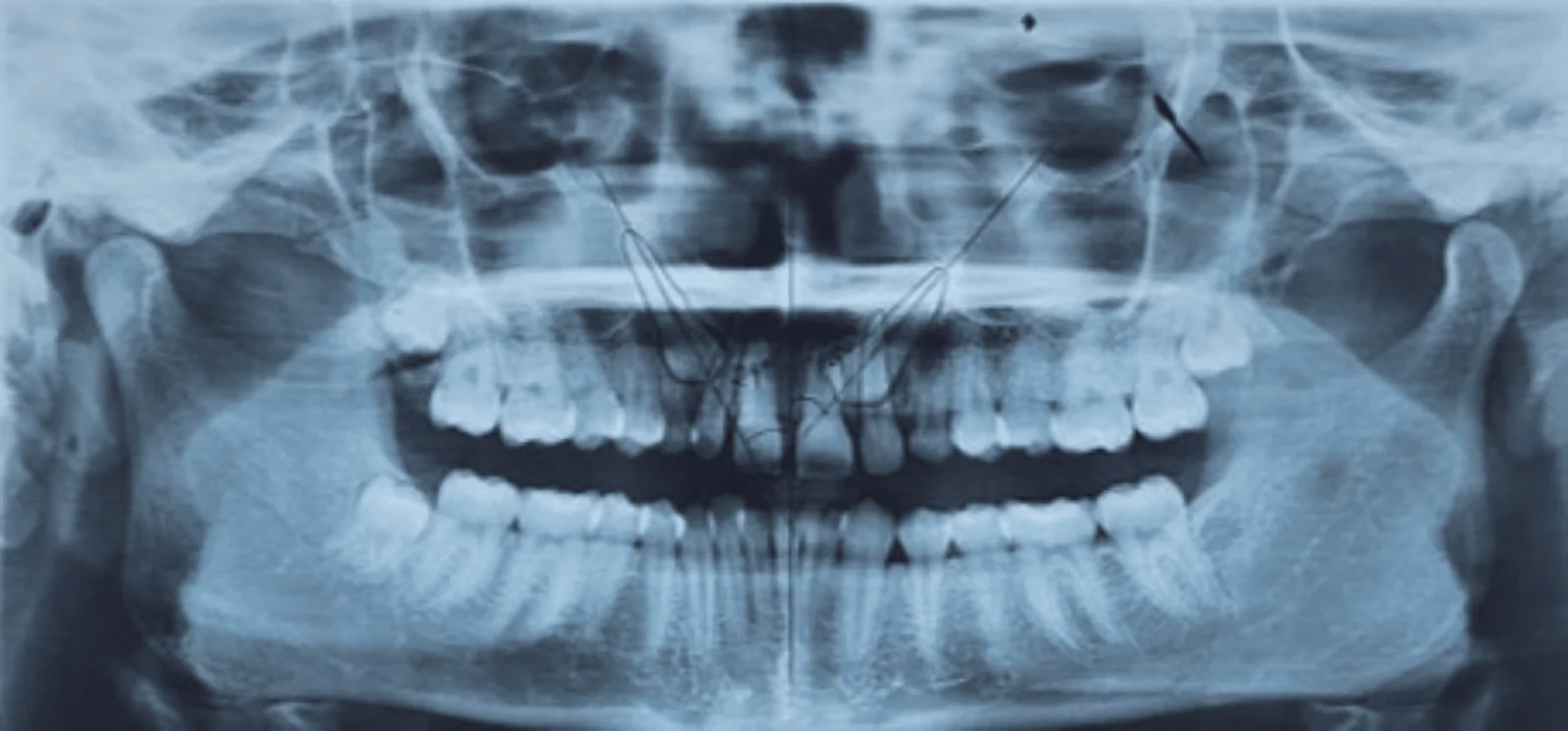
Radiographic examination. Orthopantomogram (OPG) showing the presence of bilateral palatally impacted maxillary canines and over-retained deciduous maxillary canines.

**Figure 3:**
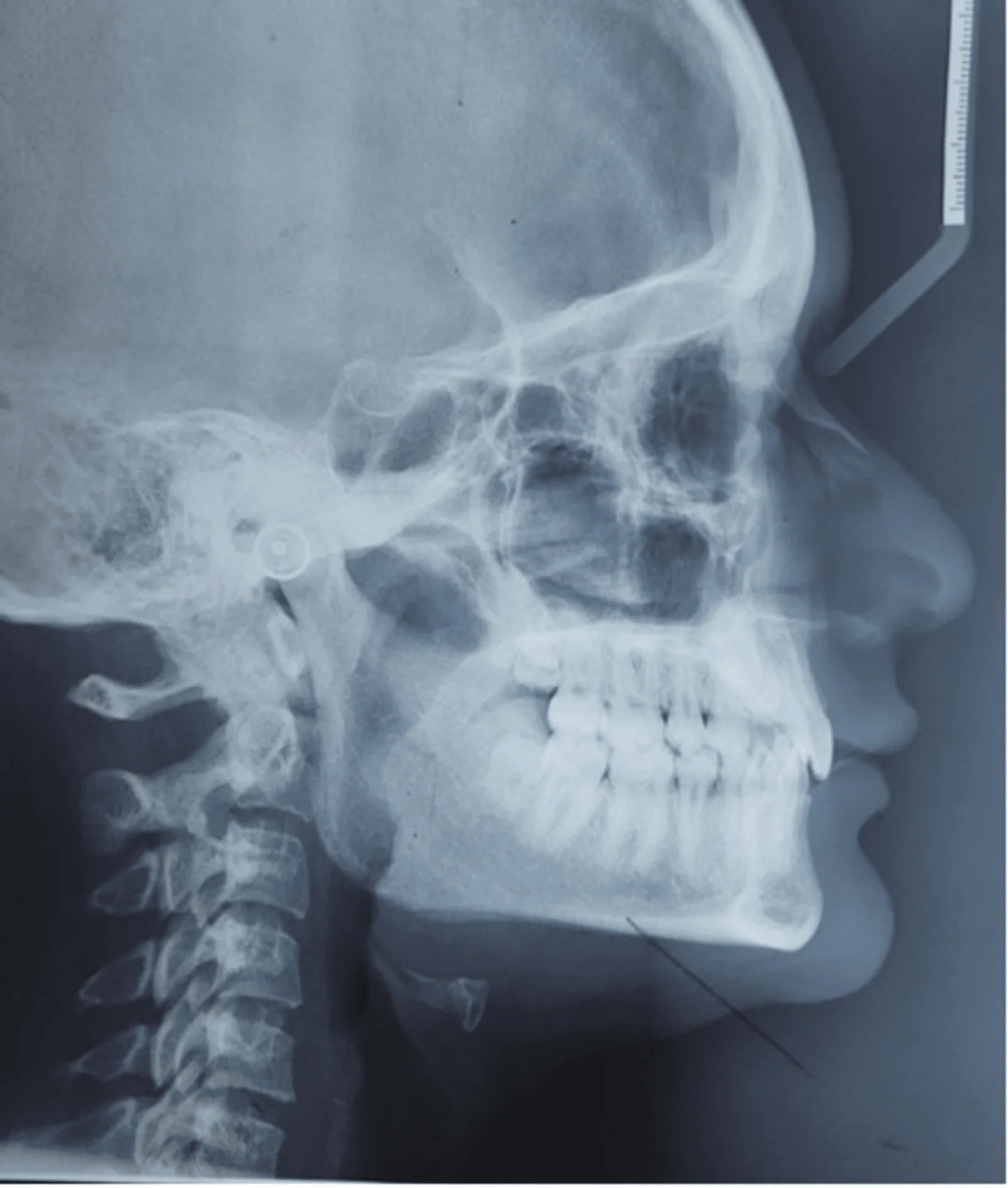
Pre-treatment lateral cephalogram.

**Figure 4:**
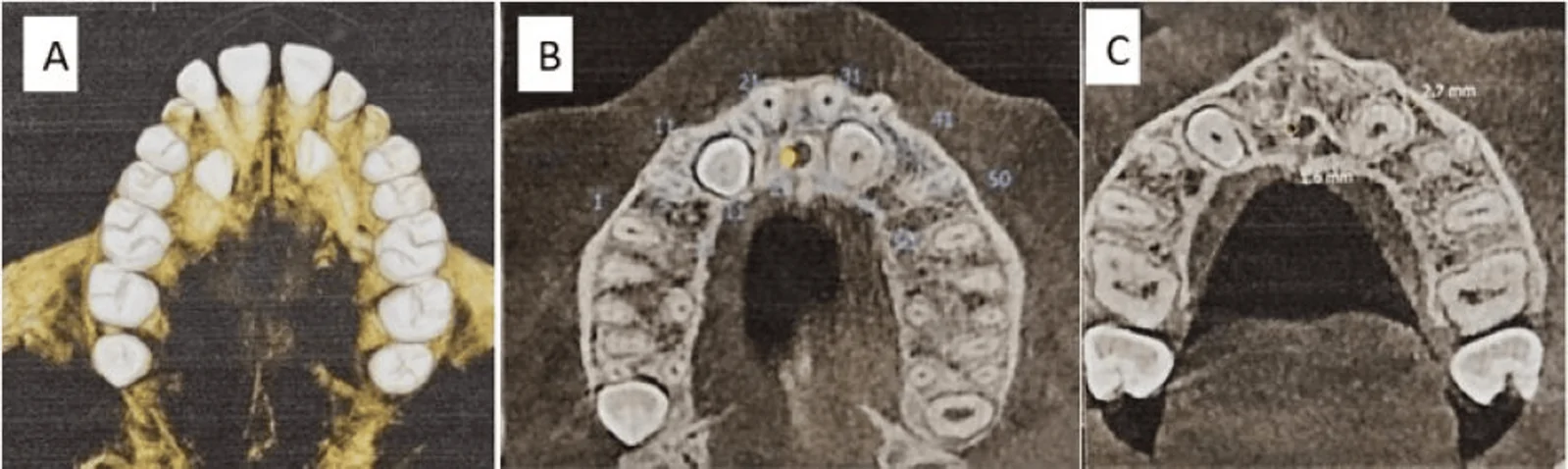
Pre-treatment cone-beam computed tomography (CBCT).

Surgical exposure technique

Following completion of the initial orthodontic alignment and establishment of adequate space for the impacted maxillary canines, minimally invasive surgical exposure was planned based on the clinical and radiographic localization of the impacted teeth. The exact position of the canine crown was determined preoperatively using CBCT imaging and clinical bone sounding. Based on these findings, the anticipated position of the crown was transferred to the palatal mucosa. The surgical exposure field was then marked cautiously in a circular fashion with an eosin pencil, which reflected the approximate size of the underlying canine crown. This specific incision was to restrict the surgical procedure to the area that was directly over the affected tooth.

The soft tissue overlying the affected canine in the premarked circular area was removed under local anesthesia and proper isolation with an electrosurgical cautery unit. This was done by removing the tissue in a conservative and incremental manner until the crown of the affected canine was sufficiently exposed. The electrosurgical approach allowed precise removal of the overlying soft tissue with minimal surgical intervention, utilizing the open window technique. Once the crown of the tooth was sufficiently exposed, the area was thoroughly examined and was ready to receive orthodontic attachments. An orthodontic lingual button was then bonded to the exposed enamel of the affected canine via a proper bonding protocol. This button was then attached to the orthodontic traction apparatus (ballista spring) to allow the impacted canine to be relocated in the dental arch. The surgical procedure that involves preoperative localization, circular placement of the surgical site, electrosurgical soft-tissue excision, exposure of the affected canine crown, orthodontic attachment placement, post-surgical observation of the surgical site to heal, and controlled orthodontic traction with the use of a ballista spring is shown (Figure 5).

**Figure 5:**
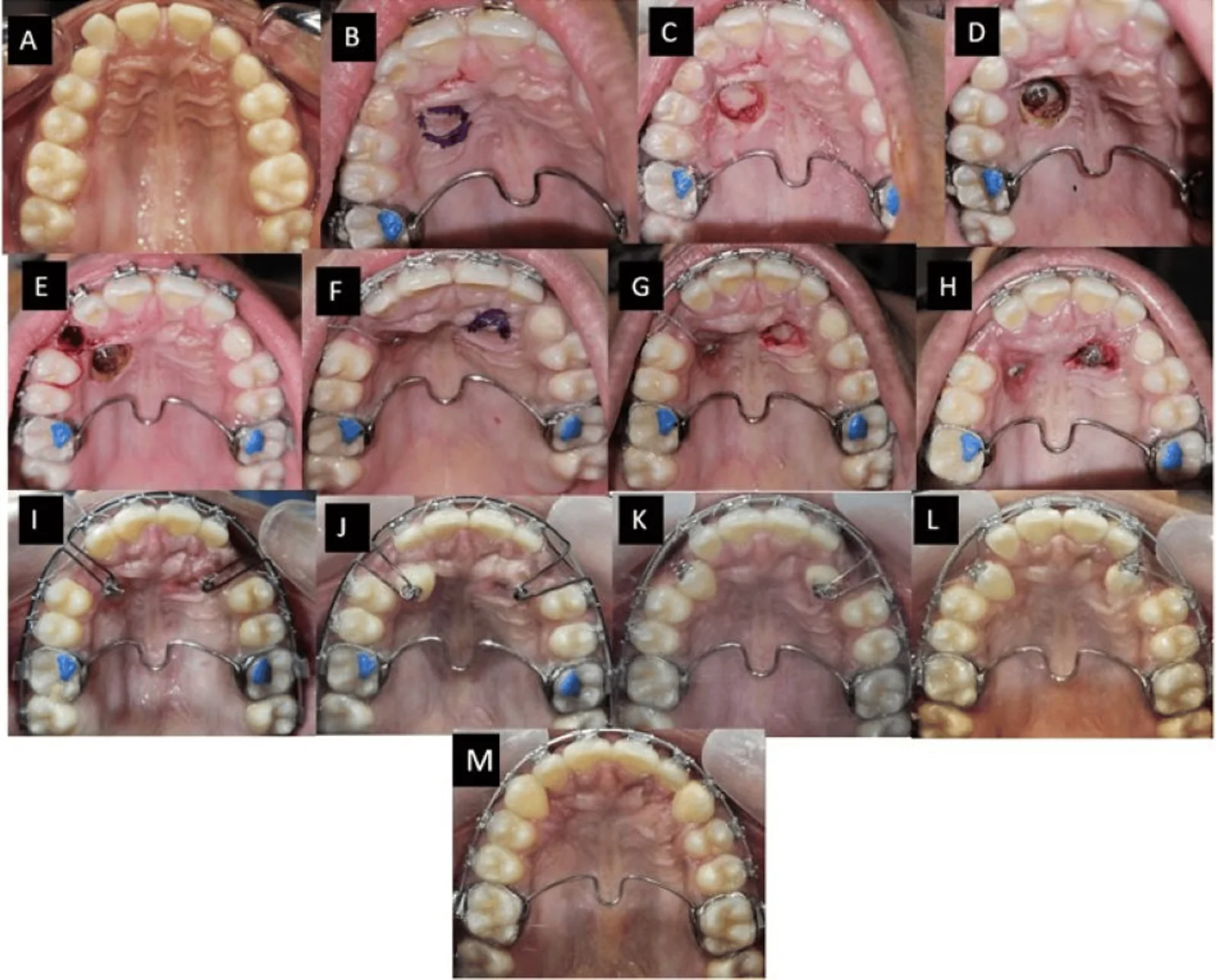
Clinical sequence demonstrating the surgical exposure and orthodontic relocation of the bilateral palatally impacted maxillary canines. (A) Pre-treatment maxillary occlusal view showing retained deciduous maxillary canines and the clinical presentation before surgical exposure. (B) Preoperative localization and circular marking of the anticipated position of the impacted right maxillary canine on the palatal mucosa based on CBCT findings and clinical bone sounding. (C) Minimally invasive electrosurgical removal of the palatal soft tissue overlying the impacted right maxillary canine, with exposure of the canine crown (open window technique). (D) Exposed canine crown following electrosurgical tissue removal with placement of an orthodontic attachment for subsequent traction. (E) Clinical view showing the exposed canine and the surgical site following placement of the orthodontic attachment, followed by extraction of the right deciduous canine. (F,G) After two weeks, the procedure was repeated for the impacted left maxillary canine. (H) Healing of the palatal surgical sites following minimally invasive electrosurgical exposure and orthodontic attachments in position. (I) Initial application of ballista springs for orthodontic relocation of the impacted maxillary canines. (J) Progressive orthodontic traction using ballista springs, demonstrating movement of the impacted canines toward the dental arch. (K) Continued canine eruption and alignment with ballista spring activation on the left side and NiTi open-coil spring placement to create space for the permanent maxillary right canine. (L) Piggyback method to position the tooth. (M) End clinical stage with successful eruption and orthodontic positioning of the maxillary canines that had been previously impacted in the dental arch.

Mid-treatment radiographs were taken, which show the alignment of both canines in the maxillary arch (Figures 6-7).

**Figure 6:**
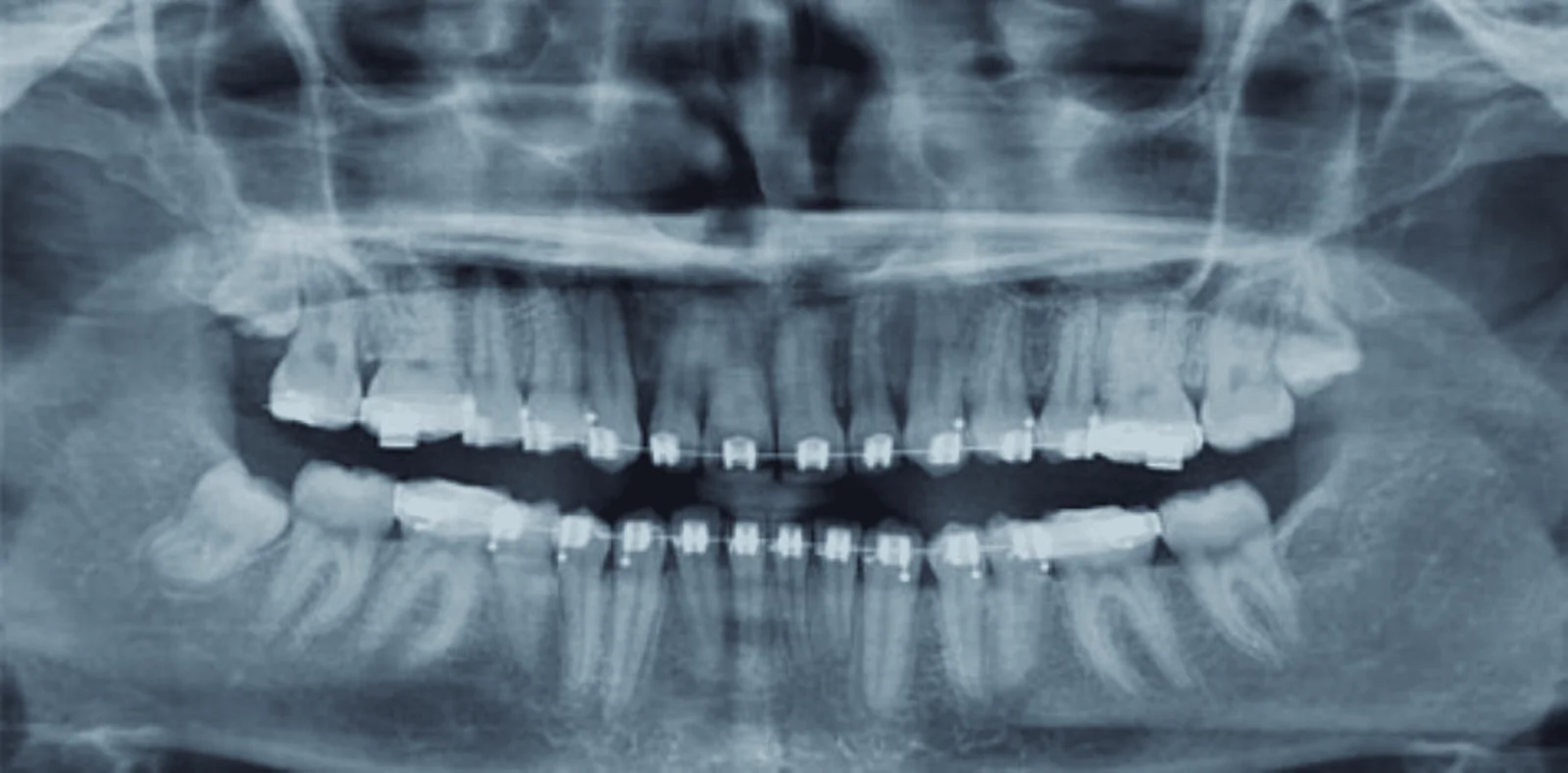
Mid-treatment orthopantomogram (OPG) showing alignment of canines in maxillary arch.

**Figure 7:**
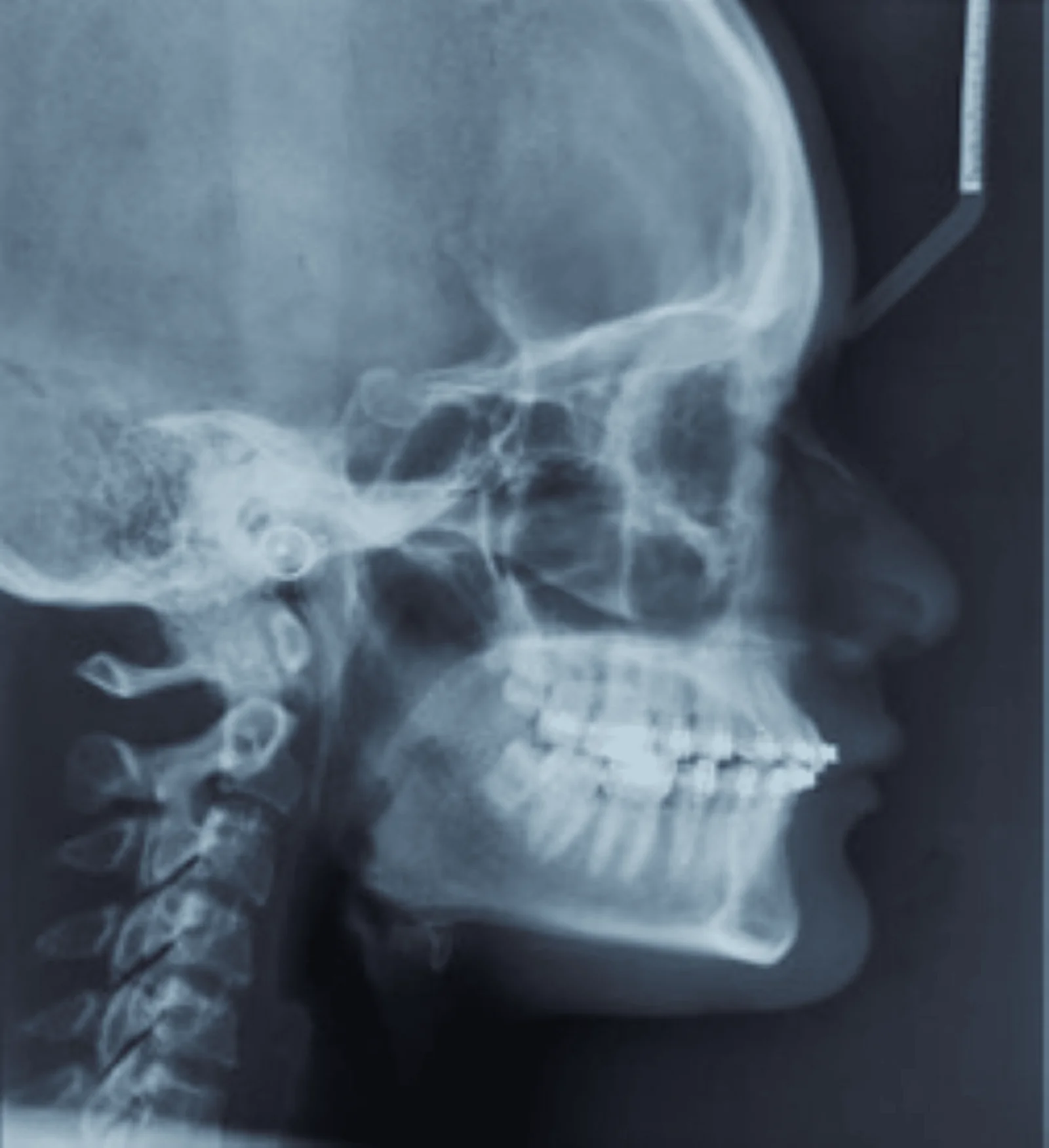
Mid-treatment lateral cephalogram.

## Discussion

Effective treatment of affected maxillary canines requires proper diagnosis, proper surgical exposure, proper space, anchorage control, and well-directed orthodontic forces [1,2]. In the current scenario, the patient experienced bilateral palatal impaction with retained deciduous maxillary canines. The alpha angle was 29° on the right and 40° on the left, indicating different degrees of canine inclination and necessitating individualized control of the direction of orthodontic traction.

The 3D location of the impacted canines was assessed with the help of CBCT, whereas clinical bone sounding assisted in determining the location of the canine crowns. These data were utilized to label the position that the crown should occupy on the palatal mucosa with an eosin pencil prior to surgery. The 3D localization has been especially applied in impacted canine management as it helps in identifying the access point of the surgery and reduces the chances of damaging neighboring teeth [4,5].

One of the peculiarities of the current case was the application of an electrosurgical exposure method that was minimally invasive. The palatal soft tissue overlying the canine crown was outlined and excised with electrosurgical cautery in a very specific circular shape rather than raising a conventional mucoperiosteal flap. Electrosurgical exposure has been reported to be an efficient technique for palatally affected canines, as it offers local tissue resection, hemostasis, and a better view of the surgical area [7]. This method in the current case allowed the direct exposure of the canine crown with minimal surgical manipulation, followed by immediate orthodontic attachment placement.

Orthodontic traction was applied to the bone after surgical exposure with a ballista spring that was made of 0.016-inch Australian stainless-steel wire possessing a high tensile strength, with a transpalatal arch as anchorage. Jacoby described the ballista spring as a simple auxiliary capable of producing controlled eruptive forces through torsional activation of the wire [8]. Raghav et al. reported successful management of bilateral palatally impacted maxillary canines using a 0.014-inch Australian wire ballista spring and transpalatal anchorage, with vertical and labial traction used to guide the canines toward the dental arch [9].

The direction of traction is critical in impacted canine treatment. An initial vertical component can help move the canine away from the palatal impaction site, after which the force vector can be modified to facilitate labial and occlusal movement into the arch [6,9]. In the present case, controlled activation of the ballista springs allowed progressive relocation of both impacted canines while maintaining posterior anchorage. The varying alpha angles of the right and left canines also highlighted the importance of precise monitoring and control of the direction of the force in the treatment.

Therefore, it can be concluded that the current case shows that accurate localization using CBCT techniques, along with clinical bone sounding, minimal exposure to electrosurgical sources, direct placement of orthodontic attachments, and a controlled ballista spring traction could offer a conservative intervention to manage bilateral palatally impacted maxillary canines. This is just one example, and the method cannot be applied to all patterns of canine impaction. However, it brings out a viable low-invasive surgical technique, which can lead to a decrease in unnecessary tissue manipulation and may result in subsequent orthodontic relocation.

## Conclusions

The ballista spring offers a flexible and efficient way of orthodontically directing palatally impacted maxillary canines. Its capacity to provide a controlled force of eruption and aid in the movement of teeth in three dimensions makes it a handy supplement in the treatment of impacted canines. To ensure favorable functional and aesthetic outcomes, early diagnosis, proper surgical exposure, prudent use of force, and routine follow-ups are critical.
